# Complete Atrioventricular Canal Defect in a Non-syndromic Adult Patient: An Unusual Presentation

**DOI:** 10.7759/cureus.43186

**Published:** 2023-08-09

**Authors:** Elias M Nabhan, Samih B Khoury, Tony E Bechara

**Affiliations:** 1 Department of Cardiovascular Medicine, Central Military Hospital, Beirut, LBN; 2 Department of Cardiology, University of Balamand, Beirut, LBN

**Keywords:** echocardiography, cyanosis, congenital heart disease, clubbing, atrioventricular canal defect

## Abstract

This case report presents a 30-year-old male patient with a history of autism spectrum disorder who experienced a drastic decline in physical activity in recent years. Upon examination, the patient was found to be relatively bedridden, lethargic, and unable to maintain an upright gait, accompanied by severe clubbing. Transthoracic echocardiography revealed a massive 4 cm complete atrioventricular canal (CAVC) defect. Although the patient was asymptomatic throughout most of his life, the CAVC defect appeared to have progressively impacted his health and activity tolerance. The importance of a multidisciplinary approach in early detection and timely intervention in managing CAVC defect is emphasized in this case.

## Introduction

Atrioventricular canal defect, also known as endocardial cushion defect, is a congenital heart anomaly characterized by abnormalities in the center of the heart involving the atrial and ventricular septa, as well as the atrioventricular valves [1]. Atrioventricular canal defect results in a large hole between the atria and ventricles of the heart, causing blood to flow freely between all four chambers. This condition is further classified into two types: complete atrioventricular canal (CACV) defect and incomplete atrioventricular canal defect [2].

In CACV defect, there is a large, single hole in the center of the heart that allows unrestricted blood flow between all four chambers, leading to significant mixing of oxygenated and deoxygenated blood. Both the atrial and ventricular septa are involved, and the atrioventricular valves are not fully developed, resulting in a more severe form of the condition [1,2].

On the other hand, incomplete atrioventricular canal defect involves a smaller hole or multiple smaller holes in the center of the heart. Although there is still some degree of blood mixing between the atria and ventricles, it is less pronounced compared to the complete form. In incomplete atrioventricular canal defect, the atrioventricular valves may be better formed than in the complete type, but they are still abnormal to some extent [3].

Both types of atrioventricular canal defect can lead to varying degrees of cyanosis and can cause symptoms such as rapid breathing, poor feeding, and fatigue. Prompt diagnosis and appropriate medical management, including surgical intervention, are crucial in managing either form of atrioventricular canal defect to improve oxygenation and prevent potential complications [4].

## Case presentation

**Figure 1:**
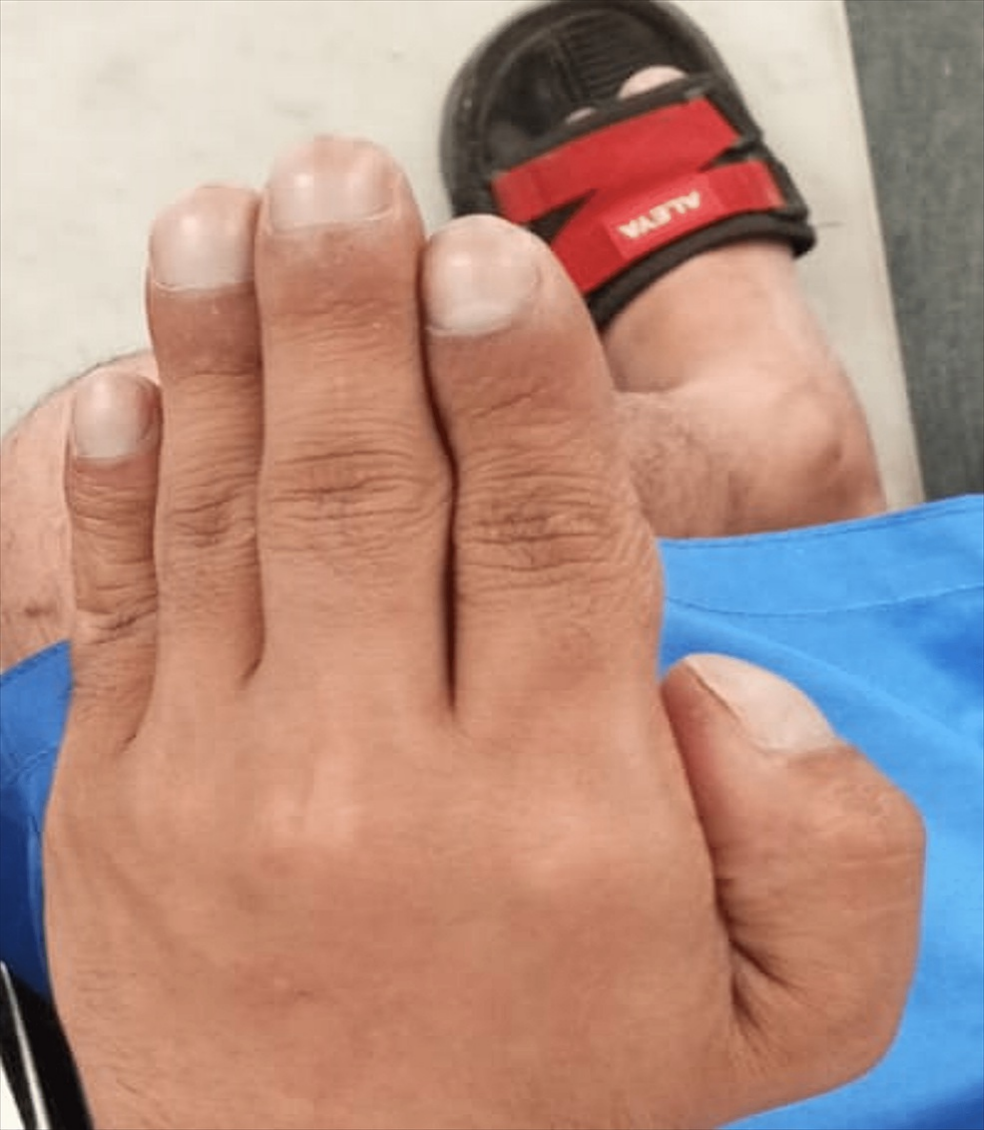
Clubbing

This is a case of a 30-year-old male patient who is a non-smoker and non-alcoholic, living in a rural area. He has a known history of autism spectrum disorder. His mother reported a significant change in his physical activity over the years. During his teenage years, he was physically active, playing with his siblings and friends, but over the last few years, he has become relatively bedridden and lethargic. He experiences an inability to maintain an upright gait and walks in a crouched position, requiring frequent rests. Concerned about his condition, his mother consulted an orthopedist who noticed severe clubbing in both upper and lower extremities (Figure 1).

Consequently, he was referred to the cardiology clinic to investigate any potential cardiac causes. A transthoracic echocardiography was performed, revealing a significant 4 cm CAVC defect with a common atrioventricular valve and moderate regurgitation. There was also a peak gradient across the right ventricle and right atria measuring 88 mmHg, along with a non-dilated compressible inferior vena cava. Additionally, moderate pericardial effusion was observed around the right ventricle without signs of tamponade. Although right ventricular hypertrophy was present, there was no dilation, and the left ventricular contractility was good. Furthermore, there was no evidence of pulmonic valvulopathies or aortic coarctation (Video 1).

**Video 1:** Transthoracic echocardiography showing apical four-chamber views (left and middle) and two-chamber view (right)

The patient and his family declined to undergo a right heart catheterization, which could have provided precise information about the presence of pulmonary hypertension and aided in further diagnostic evaluation.

Notably, the patient has one older brother and two younger sisters, all of whom do not have any congenital anomalies. Moreover, the parents are not related by blood and are in good overall health.

## Discussion

CAVC defect is a congenital heart condition characterized by an abnormality in the center of the heart involving the atrial and ventricular septa, as well as the atrioventricular valves [1]. In CAVC defect, there is a large hole that allows blood to flow freely between all four chambers of the heart, disrupting the usual separation between the left and right sides. This leads to the mixing of oxygenated and deoxygenated blood, resulting in reduced oxygen levels in the systemic circulation [1,2]. The occurrence of atrioventricular septal defect has been approximated to range from 0.24 to 0.31 per 1000 live births, with no notable disparity between males and females [1].

The causes of CAVC defect are not entirely clear, but it is believed to be associated with chromosomal abnormalities such as Down syndrome [5]. Genetic factors and certain maternal conditions may contribute to the risk of CAVC defect [6].

CAVC defect is typically detected soon after birth or during early infancy when the symptoms such as rapid breathing, poor feeding, fatigue, and failure to thrive become evident. Prompt diagnosis is crucial to initiate appropriate medical management and consider surgical intervention to repair the heart defect [1]. When left untreated, CAVC defect can lead to severe complications and negatively impact the individual’s overall health and quality of life [2]. Nevertheless, it is noteworthy that our patient remained asymptomatic throughout his life until the past few years, when a decline in physical activity became apparent, and he developed an intolerance to activities that were previously manageable for him.

As CAVC defect allows blood mixing, it is considered a cyanotic heart disease. In the presence of cyanosis, individuals with congenital heart defects, including CAVC defect, often adopt positions like crouching or squatting. This is known as the “relieving position” and is a compensatory mechanism to increase oxygenation. Crouching or squatting increases systemic vascular resistance, diverting more blood flow to the lungs for better oxygenation [7]. This could potentially explain the patient’s preference for adopting such a position.

## Conclusions

In conclusion, this case underscores the importance of early detection and intervention in congenital heart defects, especially in cases where non-cardiac symptoms or conditions may obscure the underlying cardiac issue. By carefully assessing the patient’s medical history, clinical symptoms, and diagnostic results, medical professionals can develop an optimal treatment strategy that improves the patient’s quality of life and long-term outcomes.
